# ‘Levodopa Phobia’: a review of a not uncommon and consequential phenomenon

**DOI:** 10.1038/s41531-018-0067-z

**Published:** 2018-10-02

**Authors:** Nataliya Titova, Oleg Levin, Elena Katunina, K. Ray Chaudhuri

**Affiliations:** 1 Department of Neurology, Neurosurgery and Medical Genetics, Federal State Budgetary Educational Institution of Higher Education, “N.I. Pirogov Russian National Research Medical University” of the Ministry of Healthcare of the Russian Federation, Moscow, Russia; 2 Russian Medical Academy of Professional Continuous Education, Centre of Extrapyramidal Disorders, Moscow, Russia; 3 Institute of Psychiatry, Psychology and Neuroscience at King’s College London, London, UK; 4 Parkinson Foundation International Centre of Excellence at King’s College Hospital, Denmark Hill, London, SE5 9RS UK

## Abstract

‘Levodopa Phobia’ is under-recognised in Parkinson’s disease but can cause profound detrimental clinical complications if left to continue. Several types can be encountered in clinical practice and can be driven by a misplaced fear of levodopa-induced dyskinesias, other gastrointestinal side effects and also the theoretical notion that levodopa may be toxic to dopaminergic neurons in the brain. The condition can be underpinned by a sense of strong influence from the physicians or carers who are unwilling to prescribe or consider levodopa, and also high levels of anxiety or even impulsive compulsive traits in patients who have been influenced by available literature or social media-based information. If unrecognised, the clinical issue may lead to motor deterioration and related muscle contractures leading to social isolation as well as a range of non-motor symptoms. In some, there may be emergence of intrusive impulse control disorders because of reliance on only dopamine agonists related to the fear of taking levodopa. Four cases illustrate the different patterns of ‘Levodopa Phobia’ in this study. Management of levodopa phobia is complex and includes recognition and skilled neuropsychological interventions to break the misperceptions about the complications of levodopa therapy.

## Introduction

Levodopa is the gold standard for treatment of Parkinson’s disease (PD). However, since the early 2000s, clinical guidelines for management of PD have recommended a preferential use of dopamine agonists over levodopa as initial therapy. This rationale is underpinned by several well publicised levodopa versus dopamine agonist comparative studies, which showed a delay in the development of motor complications in the dopamine agonist arm.^1–5^ In addition, dopamine agonists were favoured over levodopa because of data suggesting a ‘toxic’ effect of levodopa on nigral neuronal cell cultures, although clear evidence of levodopa toxicity in humans are lacking.^6,7^ Aggressive marketing of this concept by pharmaceutical companies, as well as neurologists and geriatricians, particularly in the early 2000 period, have led to many patients with PD to either reject the option of using levodopa even when it is clearly clinically indicated or develop a ‘fear’ about the use of levodopa. The problem was originally highlighted by Levin et al.^8^ in 2001, who described the condition of ‘Levodopa Phobia’ in PD in Russian language. His cases also highlighted rejection of levodopa intake because of problems of overemphasised fears of gastrointestinal side effects for some patients, whereas others rejected the therapy as they had an impulsive compulsive personality trait. Kurlan^9^ then reported two cases who had developed severe akinesia, because the treating neurologists were unwilling to prescribe levodopa consequent to a misplaced fear about the side effects (unacceptable and troublesome dyskinesias) and widespread publicity of the benefits of ‘levodopa sparing’ therapies. He alluded to this condition as ‘Levodopa Phobia’, a PD-specific pharmacophobia. However, recent long-term studies of levodopa versus dopamine agonist trials including the Parkinson's disease medicines trial (PDMED) study suggest otherwise and the current view is that there is little evidence to justify withholding levodopa when clinically indicated over dopamine agonists.^1,10,11^

### ‘Levodopa Phobia’ case reports

In this article we review the problem of ‘Levodopa Phobia’ among physicians, carers and patients still prevalent in real-life clinical practice and support the observations by related case reports.

#### Case 1

**Fig. 1:**
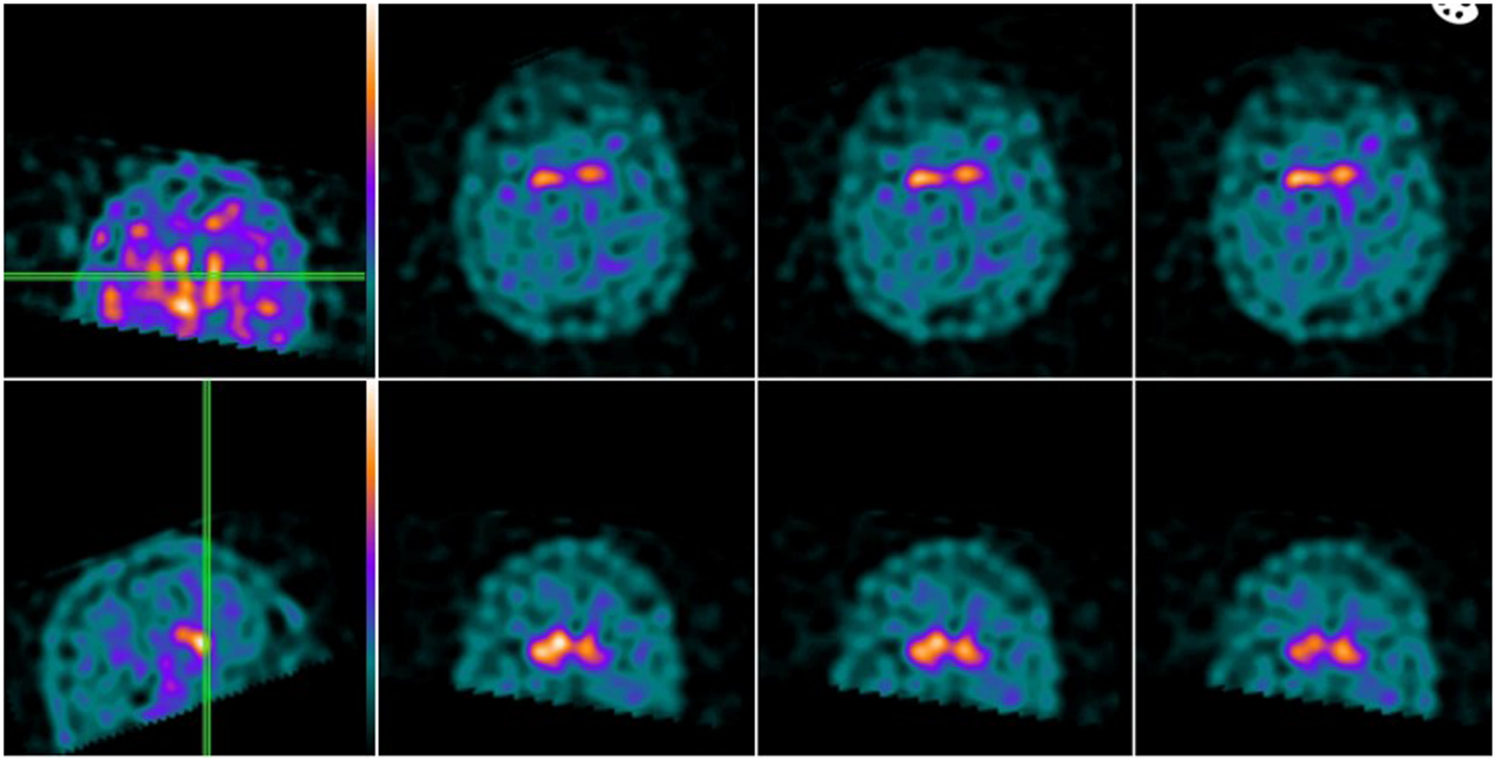
Datscan of patient (case report 1) showing bilateral loss of dopamine transporter uptake. Courtesy Nuclear Imaging Department, Kings College Hospital, London

A 45-year-old Brazilian gentleman presented to KRC at a movement disorders clinic with a potential diagnosis of ‘severe’ PD. Enquiry revealed that he developed late-onset hyposmia when aged about 35 years and also showed signs of unilateral bradykinesia about the same time. He had an introverted and anxious personality. He was seen in several clinics where initially a diagnosis of PD was considered and he was advised to take levodopa because of severe akinesia, rest tremor and a poor quality of life. However, the patient had severe anxiety about taking levodopa based on information provided by his father who also refused to allow him to try levodopa. When seen in 2016 in London, he had bilateral severe akinesia with an almost unintelligible speech, bradyphrenia, dribbling of saliva, paroxysmal rest tremor, high non-motor symptoms questionnaire score. He was ambulant on a wheelchair with upper limb contractures, although he could walk when asked. The patient and father were sceptical about dopamine loss and a Datscan was performed and confirmed severe presynaptic dopamine transporter loss with putamen-binding ratios being < 1 (right 0.64, left 0.5) (Fig. 1). ‘Levodopa Phobia’ was diagnosed and a neuropsychological ‘anti-phobia’ support programme was initiated. After several weeks the patient and the father agreed to the use of levodopa, which was started at a daily dose of 150 mg increasing to 300 mg with good motor and non-motor response. At 1-year follow-up the patient is able to perform many activities of daily living.

#### Case 2

A 48-year-old female media journalist in the United Kingdom, who was extremely well read about PD developed right-sided slowness of movement noticed when using her laptop and general slowness that was commented upon by her boyfriend and media colleagues. She also had marked fatigue and herself considered a diagnosis of PD. She then self-referred herself to KRC for further advise and management. She was felt to need immediate dopamine replacement therapy and levodopa was likely to be the most suitable drug given her bradykinesia and postural instability. However, she had severe ‘Levodopa Phobia’ being afraid about dyskinesias, which she had seen in people with PD as reported in the media and also by being member of several PD patient groups. She had also read some papers that had suggested levodopa is toxic to brain cells in animal models. As such initiation with levodopa was impossible and she was started on a dopamine agonist along with rasagiline. After 1 year she presented to the clinic with recurring bradykinesia interfering with her media work. However, even inspite of the bradykinesia and related issues she was not convinced about the need for levodopa to be started and wanted to stay on the same dose of treatment she was on. She tried various forms of complimentary medicine (herbal therapy, acupuncture, swimming and nutritional supplements) with no beneficial effect. After several consultations, a decision to use a wearable wristwatch sensor was made to document her bradykinesia in an objective manner. The report confirmed severe bradykinesia and she finally agreed to take levodopa after seeing the extent of bradykinesia but refused to go beyond 300 mg of levodopa per day. As a result, after an initial response to levodopa, bradykinesia remained a major problem and there were continuing issues related to her work in the media world.

#### Case 3

A 65-year-old Armenian teacher presented with a tremor dominant pattern of PD for 8 years. Tremor was pronounced in the left upper limb with associated pain. According to her she had a personality that was obsessed with making hand-made jewellery as a hobby. She was started and maintained on pramipexole slow release (3 mg) and amantadine (300 mg) by her treating physician. The physician had noted that levodopa therapy was not possible, as she was convinced that ‘levodopa destroys the body, it is a harmful drug and I will never take it’ in her own words. Prolonged and sustained use of pramipexole had caused severe impulse control disorder (ICD) and she had developed binge eating with marked weight gain approaching obesity in 2015. She also spent a large sum of money on compulsive shopping spree. She was troubled by severe depression and anxiety; however, she did not consult a neurologist, as she was travelling between Armenia and Russia. In 2016, she read that ICD could be caused by dopamine agonists and stopped all dopamine replacement therapy herself. She developed severe worsening of tremor and bradykinesia, and dopamine agonist withdrawal syndrome. She was then seen by NT with persisting and severe ‘Levodopa Phobia’. A trial of rotigotine transdermal patch was suggested based on data that rotigotine might be less likely to cause severe ICD but she was unable to afford the treatment. Subsequently, she was convinced to take a small dose of ropinirole (8 mg) with re-introduction of amantadine (300 mg) and addition of escitalopram (10 mg). Her ICD resolved. However, she continued to be troubled by her tremor, which she described as ‘horrible’, although objective examination revealed a low amplitude tremor likely to be helped by levodopa therapy. Inspite, she now wants to consider gamma-knife treatment for her mild tremor rather than use levodopa.

#### Case 4

NT conducted a domestic consultation to review a Russian patient who was 80 years old with PD for 14 years. He was demented, with contractures and immobile with bed sores. He was not on any dopaminergic therapy as the decision to treat was dictated by his daughter, a non-practising medical graduate with a very strong personality. Her opinion was influenced by the outcome of a consultation with a neurologist in the past, who had advised not to initiate treatment with levodopa as the drug would inevitably cause ‘problems’. Subsequently, the patient had developed hallucinations as well as confusional episodes while on dopamine agonists following which the daughter stopped dopamine agonist therapy. The daughter also did not consider another neurological consultation, as she felt that none of the available therapies could help her father and, therefore, she elected to leave him untreated. At current consultation with NT, an offer of palliation with levodopa therapy was discussed but not accepted.

## Discussion

Our cases illustrate the phenomenon of ‘Levodopa Phobia’ also referred to as pharmacophobia and is an irrational belief with consequent deleterious clinical effects in PD. The cases also suggest that several specific types of ‘Levodopa Phobia’ exist and this is also reflected by our own personal clinical experience from running large-scale PD clinics (Table 1). We believe that ‘Levodopa Phobia’ can be broadly of two types, the first being patient driven and the second related to extrinsic factors such as being driven by physicians where the issue is more of ‘aversion’ to levodopa and is described in the reports of Kurlan in addition to carer influences.^9^ In the PD patient group, ‘Levodopa Phobia’ can also be classed as an anxiety disorder related to a specific phobia about taking levodopa preparations. Patient-based ‘Levodopa Phobia’ could be of several types and can range from complete aversion to taking levodopa containing medications to secretly being non-compliant or refusing to increase the dose is inspite of clinical advice. Some patients my be afraid of levodopa use because of a potential risk of development of skin melanoma, whereas in others fear of gastrointestinal side effect is dominant. Cases observed by OL also demonstrate the peculiar phenomenon of aversion to levodopa in favour of invasive stereotactic brain surgery in the form of deep brain stimulation of subthalamic nucleus and indeed our case 3 reported by NT had considered gamma-knife therapy for her PD tremor even before considering levodopa. From a neuropsychological perspective, magical thinking on the part of the patient with PD is another factor as some patients avoid transitioning to levodopa treatment as its use denotes a new chapter in their life with PD. This may represent a concession to the disease or stark evidence that they really do have a progressive neurodegenerative disorder. In addition, we have also proposed a carer-based ‘Levodopa Phobia’, which can severely affect the patients as shown in our cases 1 and 4.

**Table 1:** Proposed patterns of ‘Levodopa Phobia’ in Parkinson’s disease

Type of subject and personality	Pattern of ‘Levodopa Phobia’	Evidence base
Primary: patient based:		
True ‘Levodopa Phobia’ (type 1)	Often associated with high anxiety states, misperception of medical data (media-based lay information on dyskinesias and side-effect profile of levodopa as provided in drug information leaflet) and complete aversion to levodopa use inspite of serious health-related consequences.	Case reports 2 and 3
‘Levodopa Phobia’ leading to poor compliance with prescribed levodopa (type 2)	Patients usually can be persuaded to start on levodopa but discontinues treatment or refuses to escalate the dose beyond a certain limit inspite of advice otherwise. Some could refrain from use of levodopa in secret as well.	Case report 2
Secondary and extrinsic factors based:		
Physician based	Usually related to misperception of knowledge of the potential risk of levodopa -induced dyskinesias. In addition, some may be influenced by data from animal models about the toxic effect of levodopa.	(Ref. ^9^), Case report 4
	The tendency to avoid levodopa could be pronounced by local clinical guidelines espousing preferential use of ‘levodopa sparing’ therapies and peer group pressure.	
Carer based	Carer attitude towards levodopa use is reflected by a negativity or complete aversion towards using levodopa in patients.	Case reports 1 and 4

### Clinical implications and observations

The true prevalence of ‘Levodopa Phobia’ either in specific countries where use of ‘levodopa sparing’ strategies are widespread or globally is unknown. Availability of conflicting information on the pros and cons of using levodopa in social media can also bias the patients’ perception of treatment and reinforce the pharmacophobia, as well as incorrect interpretation of the physicians’ recommendations. Although physician and carer-driven ‘Levodopa Phobia’ may be relatively uncommon, most patients tend to belong to type 1 and 2 variants (see Table 2). However, Type 1 with severe ‘Levodopa Phobia’ is less frequent and the one with potentially serious consequences if the condition is allowed to continue. A potential list of complications of ‘Levodopa Phobia’ compiled from our own clinical experience is listed in Table 2. In particular, intrusive ICD related to compensatory overuse of single or multiple dopamine agonists because of ‘Levodopa Phobia’ may be a particular problem as is the worsening motor state with varying non-motor features.

**Table 2:** Types of ‘Levodopa Phobia’ types and possible motor and non-motor complications

Types of ‘Levodopa Phobia’	Patterns of complications and clinical consequences
Complete ‘Levodopa Phobia’	Severe akinesia causing lack of mobility
	Contractures
	Respiratory tract infections
	Severe nociceptive pain
	Depression and anhedonia
	Social withdrawal and carer dependence
Poor compliance and secret rejection of levodopa use	Report of lack of any effect of levodopa inspite of apparent use
	Precipitation of unnecessary investigations for atypical ‘levodopa-resistant’ parkinsonism
	A tendency to seek multiple medical consultations owing to ‘lack of effect’ of therapy
	Possibility of development of impulse control disorders because of potential overuse of other (e.g., dopamine agonist) therapies

### Diagnostic considerations

Diagnosis of ‘Levodopa Phobia’ is clinical and there may be certain pointers but there are no clear guidelines. These include the presence of a high anxiety state and hypochondriasis, over-reliance on ‘alternative’ treatment strategies, self-declaration that ‘levodopa does not work or is causing side effects’ without any spousal, or partner-based corroboration. Awareness of the problem of ‘Levodopa Phobia’ among clinicians including specialists as well as carers is an unmet need. Cultural perceptions are also important as within some ethnic groups the diagnosis of PD is considered a taboo and, as such, the use of medical therapies is discouraged, while families may resort to unconventional ‘traditional’ therapies.

### Management

**Fig. 2:**
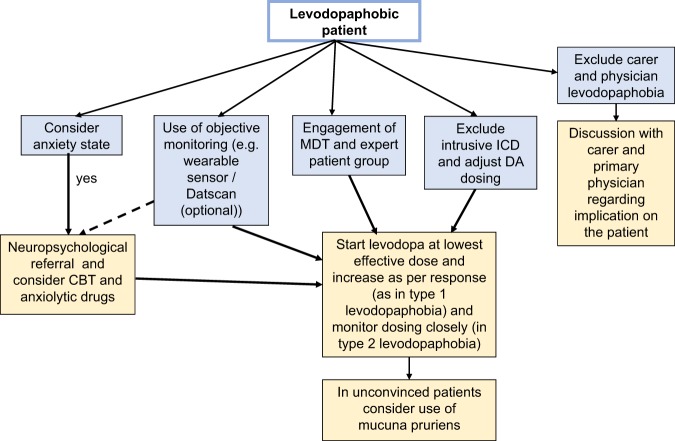
A proposed management plan of the levodopaphobic patient. MDT multidisciplinary team, CBT ognitive behavioural therapy, DA dopamine agonist, ICD = impulse control disorder

There are no guidelines for management of ‘Levodopa Phobia’ inspite of it’s potential serious consequences. Levodopa could be beneficial from early to advanced stages of PD. A visual sense of the different possible management options is proposed in Fig. 2 based on our pragmatic clinical experience. A multidisciplinary input to the problem is essential as often there is underlying severe anxiety state. In levodopaphobic patients paradoxically, anxiety can also be specifically triggered if the physician can persuade the patient to start levodopa. In these cases, emphasis must be on strategies to reduce levodopa-induced non-motor fluctuations which itself could aggravate anxiety states.^12–14^ The latter strategy is one of the ‘personality enablers’ of the recently described ‘circle of personalised medicine’ for PD.^13,14^ In selected cases, use of objective measures such as digital monitoring (wearable devices highlighting bradykinesia, Case 2) or Datscan (as in Case 1) may be helpful in convincing unwilling phobic PD patients to realise the extent of parkinsonism and the need to start levodopa therapy. Input from an expert patient group is also useful for convincing the patient regarding the benefits of levodopa therapy as is the role of a skilled psychotherapist with expertise in treating anxiety phenomena. In cases where there is severe comorbid anxiety disorder relevant anxiolytic pharmacological therapies and/or cognitive behavioural therapy may be required. If there is manifest ICD, treatment needs to be multidisciplinary with engagement of neuropsychology and neuropsychiatry teams. If one is successful in initiating levodopa therapy, patients need close surveillance for any side effects and also to ensure compliance. Anecdotally some patients have been persuaded to take mucuna pruriens (levodopa containing plant product)^15^ and subsequently switched to commercial levodopa preparation, and this may be an option worth exploring.

## Conclusions

Many phobias exists in PD patients and can range from needlephobia (relevant to subcutaneous treatments), claustrophobia (relevant to brain magnetic resonance imaging) and aversion to surgical therapy involving the brain and here we focus on a specific levodopa related phobia. Physicians may also have dopamine agonist-phobia thus refusing to prescribe any dopamine agonists when in some circumstances they can be beneficial to the patient. ‘Levodopa Phobia’ is another poorly recognised phenomenon in clinical practice sometimes with profound detrimental clinical consequences. The condition may arise from misperceptions of the side effects of levodopa by the patient but also can be precipitated by physicians or misguided but well meaning relatives inspite of the fact that levodopa remains the most effective treatment option even in the late stages of PD.^16^ Patients may suffer from underlying high anxiety state and depression aggravated by information circulating in social media. Early recognition is crucial as treatment can be time consuming and has to be multidisciplinary. There could also be cultural and ethnic differences which may drive ‘Levodopa Phobia’ in PD and large-scale epidemiological data capture is required.

## References

[1] Gray R (2014). Long-term effectiveness of dopamine agonists and monoamine oxidase B inhibitors compared with levodopa as initial treatment for Parkinson’s disease (PD MED): a large, open-label, pragmatic randomised trial. Lancet.

[2] Parkinson Study Group. (2000). Pramipexole vs levodopa as initial treatment for Parkinson disease: a randomized controlled trial. JAMA.

[3] Rascol O (2000). A five-year study of the incidence of dyskinesia in patients with early Parkinson’s disease who were treated with ropinirole or levodopa. N. Engl. J. Med..

[4] Oertel WH (2006). Pergolide versus levodopa monotherapy in early Parkinson’s disease patients: The PELMOPET study. Mov. Disord..

[5] Rinne UK (1998). Early treatment of Parkinson’s disease with cabergoline delays the onset of motor complications. Results of a double-blind levodopa controlled trial. The PKDS009 Study Group. Drugs.

[6] Fahn S (1997). Levodopa-induced neurotoxicity: does it represent a problem for the treatment of Parkinson’s disease?. CNS Drugs.

[7] Fahn S, Cohen G (1992). The oxidant stress hypothesis in Parkinson’s disease: evidence supporting it. Ann. Neurol..

[8] Левин ОС (2001). Лечение болезни Паркинсона на ранней стадии. В мире лекарств..

[9] Kurlan R (2005). “Levodopa phobia”: A new iatrogenic cause of disability in Parkinson disease. Neurology.

[10] Katzenschlager R (2008). Parkinson’s Disease Research Group of the United Kingdom. 14-year final report of the randomized PDRG-UK trial comparing three initial treatments in PD. Neurology.

[11] Parkinson Study Group CALM Cohort Investigators. (2009). Long-term effect of initiating pramipexole vs levodopa in early Parkinson disease. Arch. Neurol..

[12] Storch A (2013). Nonmotor fluctuations in Parkinson’s disease: severity and correlation with motor complications. Neurology.

[13] Titova N (2017). The future of Parkinsonas treatment - personalised and precision medicine. European Neurological Review.

[14] Titova N, Chaudhuri KR (2018). Non-motor Parkinson disease: new concepts and personalised management. Med J. Aust..

[15] Cassani E (2016). Mucuna pruriens for Parkinson’s disease: low-cost preparation method, laboratory measures and pharmacokinetics profile. J. Neurol. Sci..

[16] Rosqvist K (2018). Levodopa effect and motor function in late stage Parkinson’s disease. J. Park. Dis..

